# Cigarette smoke reduces short chain fatty acid production by a *Porphyromonas gingivalis* clinical isolate

**DOI:** 10.1111/jre.12660

**Published:** 2019-04-14

**Authors:** Iris Zeller, Marina V. Malovichko, Harrell E. Hurst, Diane E. Renaud, David A. Scott

**Affiliations:** ^1^ Departments of Oral Immunology and Infectious Diseases University of Louisville Louisville Kentucky; ^2^ University of Louisville Superfund Research Center and Envirome Institute, University of Louisville Louisville Kentucky; ^3^ American Heart Association Tobacco Regulatory Science and Addiction Center University of Louisville Louisville Kentucky; ^4^ Pharmacology and Toxicology University of Louisville Louisville Kentucky; ^5^ Present address: Division of Clinical Microbiology, Department of Laboratory Medicine Medical University of Vienna Vienna Austria

**Keywords:** chronic periodontitis, *Filifactor alocis*, *Fusobacterium nucleatum*, *Porphyromonas gingivalis*, short chain fatty acids, tobacco smoking

## Abstract

**Objectives:**

We hypothesized that short chain fatty acid (SCFA) production by oral pathogens is suppressed by exposure to cigarette smoke extract (CSE).

**Background:**

Tobacco smoking is a major risk factor for plaque‐induced periodontal diseases. Despite increased disease susceptibility, overt oral inflammation is suppressed in smokers, presenting a diagnostic conundrum. Bacterial‐derived SCFAs can penetrate into oral tissues where they influence multiple components of immune and healing responses. Indeed, the SCFA burden has been correlated with the inflammatory condition of the gingiva. However, the influence of cigarette consumption on SCFA production is unknown.

**Methods:**

GC/MS was employed to monitor the production of several SCFAs (propionic acid, isobutyric acid, butyric acid, and isovaleric acid) by representative anaerobic oral pathogens (*Filifactor alocis* 35896, *Fusobacterium nucleatum* 25586, *Porphyromonas gingivalis* 33277) that were exposed, or not, to a physiologically relevant dose of CSE (2000 ng/ml nicotine equivalents) generated from 3R4F reference cigarettes.

**Results:**

The growth of all three bacterial species was unaffected by CSE. The capacity to produce SCFAs by these bacteria was highly varied. *F alocis* produced the highest concentration of a specific SCFA (butyrate); *P gingivalis* provided the most robust overall SCFA signal, while *F alocis* and *F nucleatum* did not release detectable levels of isobutyrate or isovalerate. As *P gingivalis* 33277 was the broadest SCFA producer, three low‐passage clinical isolates (10208C, 5607, and 10512) were also examined. Compared to unconditioned microbes, reduced SCFA release was apparent in CSE‐exposed low‐passage clinical isolates of *P gingivalis* which reached significance for one of the three isolates (propionic, isobutyric, butyric, and isovaleric acids, all *P* < 0.05).

**Conclusions:**

There is high disparity in the SCFA profiles of variant chronic periodontitis‐associated bacteria, while CSE exposure reduces SCFA production by a specific clinical strain of *P gingivalis*. If the latter phenomenon occurs in vivo, a reduced SCFA burden may help explain the reduced vascular response to dental plaque in tobacco smokers.

## 1 INTRODUCTION

Cigarette use is a major risk factor for chronic periodontitis and other periodontal diseases.^1^ Smoking has been shown to increase susceptibility to destructive disease and to promote colonization by several pathogens, including *Porphyromonas gingivalis* and *Fusobacterium nucleatum*.^1^, ^2^ Smoking also reduces the efficacy of multiple treatment modalities like scaling and root planing 3; surgical interventions 4; adjunctive antimicrobial therapy 5, 6; and bone regeneration efforts,^7^ while smoking cessation has a positive impact on the periodontal tissues.^8^, ^9^, ^10^, ^11^ There is also increasing evidence that the ill effects of smoking on periodontal health are dose‐related 12 and that environmental tobacco smoke exposure may predispose to disease.^13^, ^14^, ^15^, ^16^, ^17^ Interestingly, gingival bleeding—an important clinical indicator for the presence of periodontal diseases—is suppressed in smokers. As we have recently reviewed, this discrepancy can be attributed to suppressed gingival angiogenesis in response to plaque.^18^ In a previous study, we were also able to show that *P gingivalis* becomes a less potent inducer of pro‐inflammatory responses from mononuclear cells upon exposure to cigarette smoke extract (CSE). This decrease in inflammatory potential could be due to differential regulation of several genes in CSE‐treated bacteria; for example, genes involved in the biosynthesis of the inflammatory capsule were downregulated.^19^ A reduced inflammatory response would be expected to facilitate the persistence of *P gingivalis* in its subgingival niche. However, the mechanisms of tobacco‐induced and/or tobacco‐exacerbated chronic periodontitis remain to be clarified.

Short chain fatty acids (SCFAs) are common metabolic products of bacteria. There are multiple potential mechanisms by which increased SCFA production could contribute to disease progression in a general population.^20^, ^21^, ^22^, ^23^ For example, SCFAs and other bacterial fermentation end products have been shown to drive epigenetic changes in eukaryotic cells 24 with negative effects on epithelial cell,^25^ fibroblast,^26^, ^27^ and T‐cell 28, 29 reproduction noted. Furthermore, SCFA has been shown to activate NF‐κB and stimulate pro‐inflammatory cytokine release 30, 31, 32; attract innate immune cells 33; promote vascular dilation 32; and endothelial proliferation.^32^, ^34^ SCFAs have been ascribed antimicrobial properties against some bacteria,^35^, ^36^ while they promote biofilm formation by others, including *Actinomyces naeslundii*.^37^ Thus, it is possible that alterations to the SCFA status quo, increases or decreases, could contribute to the microbial flux in subgingival plaque. SCFA concentrations in the gingival crevice correlate with the severity of inflammation, including periodontal bleeding and elevated subgingival temperature.^34^, ^38^

Because increased susceptibility to chronic periodontitis in tobacco users is accompanied by a decreased vascular response (ie, bleeding on probing, but also decreased production of gingival crevicular fluid) to dental plaque, we hypothesized that exposure to CSE may suppress SCFA production by *P gingivalis* and other relevant pathogens. Such reduced SCFA release would be consistent with the reduced vascular and inflammatory indices, including the suppressed bleeding response observed in human smokers with periodontal diseases.

To the best of our knowledge, the influence of smoking on SCFA production by oral pathogens has not been addressed. Thus, we aimed to evaluate levels of SCFAs (propionic acid CH_3_CH_2_COOH, isobutyric acid (CH_3_)_2_CHCOOH, butyric acid CH_3_(CH_2_)_2_COOH, and isovaleric acid (CH_3_)_2_CHCH_2_COOH) in culture supernatants of two established major SCFA‐producing gram‐negative pathogens, *P gingivali*s and *F nucleatum*,^28^ as well as the emerging gram‐positive pathogen, *Filifactor alocis* exposed to CSE.

## 2 MATERIAL AND METHODS

### 2.1 Materials

Gifu Anaerobe Medium was purchased from Nissui Pharmaceutical, Tokyo, Japan; brain heart infusion (BHI) came from Beckton Dickinson (Sparks, MD); 3R4F standard reference cigarettes from the Kentucky Tobacco Research and Development Center, Lexington, KY; dichloromethane came from Honeywell, Morristown, NJ; analytical standards for propionic, butyric, isobutyric, and isovaleric acid, sodium butyrate‐^13^C_4_, Na_2_SO_4_, menadione, hemin, arginine, and cysteine were purchased from Sigma‐Aldrich, St. Louis, MO; nicotine D_4_ came from Cerilliant, Round Rock, TX; NaOH, and HCl from Fisher Scientific, Pittsburgh, PA; fetal bovine serum (FBS) was bought from Atlanta Biologicals (Lawrenceville, GA, USA); helium was obtained from Welders Supply (Louisville, KY); while N‐Methyl‐N‐tert‐butyldimethylsilyltrifluoroacetamide (MTBSTFA) came from Regis Technologies, Morton Grove, IL.

### 2.2 Bacterial culture

*Porphyromonas gingivalis* 33277, *F nucleatum* 25586, and *F alocis* 35896, as well as the clinical *P gingivalis* isolates 10512, 5607, and 10208C from our own collection, were revived from frozen stocks. Briefly, the clinical isolates were obtained by sampling the subgingival microbiota of subjects with periodontitis with filter paper strips placed in deep periodontal pockets for 30 seconds. Microbiota was cultured anaerobically on blood agar containing gentamicin, and black‐pigmented colonies were screened with 16S rRNA probes specific for *P gingivalis*. Bacteria were grown in CSE‐conditioned and control GAM, BHI (supplemented with 5 μg/ml hemin and 1 μg/ml menadione), or BHI (supplemented with 0.1% cysteine, 20% arginine, 5% fetal bovine serum), respectively, under anaerobic conditions at 37˚C in a Coy Laboratories (Grass Lake Charter Township, MI) anaerobic chamber.

### 2.3 Preparation of CSE

CSE‐conditioned media were prepared using 3R4F reference cigarettes, as previously described 19 and employed at the physiologically relevant concentration of 2000 ng/ml nicotine equivalency,^39^, ^40^ as determined by gas chromatography–mass spectrometry (GC/MS). Briefly, 500 µl aliquots of analytical nicotine standards in control media (0‐20 000 ng/ml) were mixed with 20 µl nicotine D_4_ (final concentration 3846 ng/ml) and brought to pH of 13.0 by addition of 100 µl 2M NaOH. For the extraction of nicotine, 600 µl dichloromethane was added, samples were vortex‐mixed for 5 seconds, and layers allowed to separate. The organic phase was transferred into disposable glass cell culture vials containing the drying agent sodium sulfate. About 100 µl of the dehydrated solution was transferred into autosampler vials with low volume inserts. About 1 µl per sample was injected into the gas chromatograph for separation and nicotine concentration in CSE‐conditioned media determined by comparison to the standards.

### 2.4 Analysis of SCFA concentrations

Supernatants (14 000 g, 4˚C, 3 minutes) from late log or early stationary phase bacterial cultures were filtered through 0.2 µm filters and diluted 1/500 in deionized water prior to SCFA analysis. SCFAs were extracted from 1 ml culture supernatant aliquots with 10 µl sodium butyrate‐^13^C_4_ used as an internal standard. Samples were mixed with 5 µl 0.5 mol/L HCl and 1 ml dichloromethane. Layers were allowed to separate, and the organic phase was transferred into disposable glass cell culture vials containing sodium sulfate. About 100 µl of the dehydrated solution was transferred into autosampler vials with low volume inserts. Tert‐butyl dimethylsilyl (TBDMS) derivatives were allowed to form after the addition of 5 µl MTBSTFA for 20 minutes at 80˚C. About 1 µl of the mixture was injected into the gas chromatograph for separation. A model 5973 Hewlett‐Packard gas chromatography/mass spectrometry system with a 15 m × 0.25 mm DB‐5 UI column (Agilent Technologies, Santa Clara, CA) was used. The injection mode was splitless, oven temperatures were programmed from 60˚C to 120˚C at 10˚C/min after a 1 minute hold at the initial temperature, and, after a 1 minute hold at 120˚C, to 280˚C at 25˚C/min, with a 2 minutes hold at the final temperature. Helium was used as the carrier gas with a constant flow rate of 1.0 ml/min. All experiments were carried out in triplicate, unless otherwise stated.

### 2.5 Statistics

All statistical analyses were carried out using instat v3.06 (GraphPad, La Jolla, CA). Experiments were performed in triplicates. Differences in bacterial growth as well as interstrain variation in SCFA production were determined by ANOVA, while intrastrain variation in SCFA levels was determined by *t* test.

## 3 RESULTS

**Figure 1:**
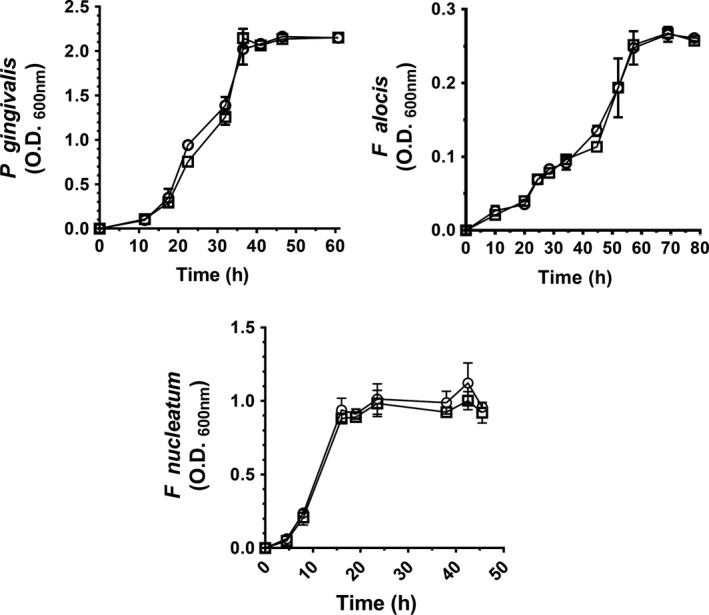
Cigarette smoke extract does not adversely influence the growth of oral pathogens. *P gingivali*s 33277 (top left), *F nucleatum* (top right), and *F alocis* (bottom) growth in control (○) and CSE‐conditioned (2000 ng/ml nicotine equivalents) media (□) was monitored spectrophotometrically at OD 600_nm_. Data are presented as mean values ± standard deviations. CSE exposure did not have a statistically significant influence on bacterial growth characteristics, as determined by ANOVA

There were no significant differences in the growth characteristics of any bacterium in CSE‐conditioned versus control media (all *P* > 0.05), as measured spectrophotometrically at OD 600_nm_ and as presented in Figure 1. While we have previously shown that *P gingivalis* can withstand high doses of CSE,^19^, ^41^, ^42^ this is the first report that *F alocis* and *F nucleatum* are tolerant to the complex mixture of toxins present in CSE, at least as assessed by replicative capacity.

**Figure 2:**
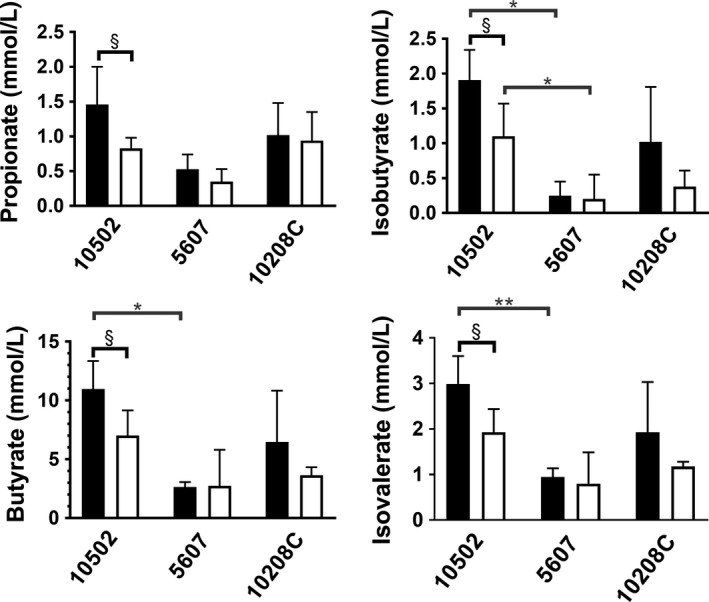
Cigarette smoke suppresses the production of SCFAs in *P gingivalis* 10502. Propionic acid (top left), isobutyric acid (top right), butyric acid (bottom left), and isovaleric acid (bottom right) production by *P gingivali*s 10502, 5607, and 10208C grown in control (black bars) or CSE‐conditioned (2000 ng/ml nicotine equivalents) medium (white bars) was determined by GCMS. SCFA release by CSE‐exposed strains compared to non‐exposed control bacteria (intrastrain variation) was determined by t test. Interstrain variation in the SCFA release was determined by ANOVA. Values are presented as mean ± standard deviation. *P* < 0.05 (*^/§^) or 0.01 (^**^), respectively

As shown in Table 1, the SCFA signal from differing oral anaerobes was highly varied. Butyric acid was the predominant SCFA released by *P gingivalis*,* F alocis,* and *F nucleatum*, alike. *F alocis* produced the highest concentration of a specific SCFA (butyrate), while *F alocis* and *F nucleatum* did not release detectable levels of isobutyrate or isovalerate. The most robust, inclusive SCFA profile was produced by *P gingivalis* 33277. Therefore, we also examined SCFA production by several low‐passage clinical isolates of *P gingivalis* (10512, 5607 and 10208C). As presented in Figure 2, SCFA production by these strains was influenced by exposure to CSE. For the isolate 10502, a significant decrease in the levels of all four SCFAs was observed upon CSE exposure.

**Table 1:** SCFA production by *P gingivalis*,* F alocis,* and *F nucleatum* species is highly variable

SCFA (mmol/L )	*P gingivalis* 33277		*F alocis* 35896		*F nucleatum* 25586	
	Control	CSE	Control	CSE	Control	CSE
Propionate	0.85 (0.20)	1.22 (0.12)^*^	6.95 (9.26)	20.13 (10.62)^#^	2.06 (1.46)	1.24 (0.39)
Isobutyrate	0.58 (0.20)^**/##^	0.46 (0.08)^***/###^	0.00 (0.00)	0.00 (0.00)	0.00 (0.00)	0.00 (0.00)
Butyrate	4.67 (1.03)^**/#^	6.02 (0.90)^**/#^	15.83 (0.70)	15.83 (0.42)	13.19 (4.41)	12.76 (3.23)
Isovalerate	1.34 (0.20)***^/###^	1.48 (0.14)***^/###^	0.00 (0.00)	0.00 (0.00)	0.00 (0.00)	0.00 (0.00)

Oral bacteria were grown with or without cigarette smoke extract (CSE; 2000 ng/ml nicotine equivalents). SCFA release by cultures in late log or early stationary phase was monitored using GC/MS (normalized to cell number). Interspecies differences in SCFA release between control or CSE‐treated cultures were determined by ANOVA. Intraspecies differences in SCFA release from control vs CSE‐exposed cultures were determined by *t* test. Values are presented as mean ± standard deviation.

*P* < 0.05 (^#/^*); 0.01 (^##/^**) or 0.001 (^###/^***), respectively, compared to *F alocis or ^#^ *F  nucleatum*.

## 4 DISCUSSION

As noted earlier, SCFAs drive vasodilation, endothelial activation, and release of pro‐inflammatory cytokines. SCFAs, including propionic, butyric, and isovaleric acids, have been negatively correlated with periodontal inflammation. However, reports on bacterial SCFA production and plaque‐induced periodontal diseases are, in some ways, controversial. For example, the literature is in disagreement over whether or not increased SCFA concentrations at diseased sites reflect increased numbers of periodontal pathogens.^21^, ^34^ Since previous reports in this area have not addressed the smoking status of the subjects, variation in tobacco use may help explain such discrepancies.

Tobacco, as we have reported previously, induces multiple transcriptomic and structural alterations in *P gingivalis* that reduce the overall inflammatory potential of this bacterium.^19^, ^41^, ^42^ We proposed that components present in CSE may also suppress SCFA production by *P gingivalis* and other oral bacteria. This hypothesis appears to be true for a specific clinical *P gingivalis* isolate, but not for the type strain, or *F nucleatum* or *F alocis*.

A major limitation of our study is that only three clinical isolates of *P gingivalis* were available for analysis; even though a trend to reduced SCFA production was noted in all three clinical isolates, significance was only reached for strain 10502. However, should this phenomenon occur in a number of clinical strains in vivo, a further mechanism by which the pro‐angiogenic, pro‐inflammatory potential of this key bacterium may be dampened in smokers is established.

Increasing evidence suggests that an imbalance in SCFA production by the gut microbiome may play a role in the development of obesity, diabetes, inflammatory bowel disease, and even colorectal cancer.^43^, ^44^, ^45^ If tobacco use has the potential to affect bacterial SCFA production, this mechanism could have profound influence beyond the oral cavity.

## CONFLICT OF INTEREST

The authors have no conflict of interest to declare.
